# Healthcare costs and productivity losses in treatment-resistant depression in Finland

**DOI:** 10.1192/j.eurpsy.2022.686

**Published:** 2022-09-01

**Authors:** S. Rannanpää, H. Taipale, A. Tanskanen, M. Lähteenvuo, S. Huoponen, J. Tiihonen

**Affiliations:** 1 Janssen-Cilag, Medical Affairs, Neuroscience, Espoo, Finland; 2 University of Eastern Finland, Department Of Forensic Psychiatry, Kuopio, Finland

**Keywords:** treatment resistant depression, healthcare utilization, cost, Depression

## Abstract

**Introduction:**

Due to its relatively high prevalence and recurrent nature, depression causes a major burden on healthcare systems and societies.

**Objectives:**

To investigate healthcare resource utilization and costs associated with treatment-resistant depression (TRD) compared with non-TRD depression in Finland.

**Methods:**

Of all patients aged 16-65 years and diagnosed with depression in Finland during 2004-2016, persons with TRD (N=15 405) were identified from nationwide registers and matched 1:1 with comparison persons with depression but no TRD. TRD was defined as initiation of a third treatment trial after having failed two pharmacological treatment trials. Follow-up period covered five years after TRD or corresponding matching data (until end of 2018). Healthcare resource utilization was studied with negative binomial regression and average excess costs of TRD with generalized estimating equations, by adjusting for baseline costs, comorbidity and baseline severity of depression.

**Results:**

Persons with TRD (mean age 38.7, SD 13.1, 60.0% women) had more healthcare utilization and work disability (sick leaves and disability pensions), adjusted incidence rate ratio for work disability days was 1.72 (95% CI 1.64-1.80). This resulted in higher total costs for persons with TRD, adjusted mean difference 7572 (95% CI 7215-7929) EUR per patient per year, higher productivity losses (due to sick leaves and disability pensions, mean difference 5296, 95% CI 5042-5550) and direct healthcare costs (2002, 95% CI 1853-2151) compared with non-TRD patients. Mean difference was highest during the first year after TRD (total costs difference 11760, 95% CI 11314-12206).

**Conclusions:**

Treatment-resistant depression is associated with a significant cost burden.

**Disclosure:**

This study was funded by Janssen-Cilag Finland and the Finnish Ministry of Social Affairs and Health through the developmental fund for Niuvanniemi Hospital. ML was partly funded by personal grants from the Finnish Medical Foundation and Emil Aaltonen fou

